# Introducing Plant-Based Mediterranean Diet as a Lifestyle Medicine Approach in Latin America: Opportunities Within the Chilean Context

**DOI:** 10.3389/fnut.2021.680452

**Published:** 2021-06-25

**Authors:** Catalina Figueroa, Guadalupe Echeverría, Grisell Villarreal, Ximena Martínez, Catterina Ferreccio, Attilio Rigotti

**Affiliations:** ^1^Centro de Nutrición Molecular y Enfermedades Crónicas, Escuela de Medicina, Pontificia Universidad Católica de Chile, Santiago, Chile; ^2^Departamento de Nutrición, Diabetes y Metabolismo, Escuela de Medicina, Pontificia Universidad Católica de Chile, Santiago, Chile; ^3^Magíster en Nutrición, Escuela de Medicina, Pontificia Universidad Católica de Chile, Santiago, Chile; ^4^Departamento de Salud Pública, Escuela de Medicina, Pontificia Universidad Católica de Chile, Santiago, Chile

**Keywords:** Mediterranean diet, plant based diet, lifestyle medicine, planetary health, chronic diseases - prevention and control, Latin America, cultural adaptability

## Abstract

Latin America is experiencing a significant epidemiological and nutritional transition, with a trend toward higher incidence of food-related chronic diseases. In this context, Lifestyle Medicine (LM) is a growing field focused on assisting individuals in adopting healthy behaviors for the prevention and treatment of these chronic diseases, including, among other pillars, a great emphasis on healthy eating. There is also a growing interest worldwide in environmental sustainability of dietary patterns, with increasing concern about their effects on planetary health. In this context, whole-food, plant-based diets -such as the Mediterranean diet (MD)- have emerged as a solution for both healthier eating and lowering environmental impact. Yet in order to be effective at these goals and achieve a high adherence to any nutritional prescription, the sociocultural reality of the community or population where we aim to practice must also be taken into account. In this review, we specifically highlight the plant-based MD as a LM-contextualized dietary pattern that is adaptable, applicable, and sustainable within the Chilean context and has the potential to address the current trend of chronic diseases in our country.

## Introduction

During past decades, the patterns of health and disease have changed, explained mostly by a demographic shift and increasing urbanization and industrialization. This has led to an epidemiological transition characterized by a decrease in mortality from infectious diseases, a rise in life expectancy, and a sustained increase in non-communicable chronic diseases (NCDs) (1–4). Latin America and developing countries are not the exception to this trend, showing a double burden of infectious and chronic diseases (5). Even more, since the beginning of this century, countries such as Chile and Uruguay have already depicted age-adjusted mortality rates from cardiovascular diseases (CVD) and cancer similar to those found in developed countries, such as the United States and Canada (6). Correlation between certain lifestyle habits, such as unhealthy diet and low physical activity, and the risk of developing NCDs is already well-known (7). Reducing diet-related risk factors associated with these conditions is one of the most impactful ways to reduce NCD burden as well as lessening their detrimental influence on individuals and society as a whole (8, 9).

The largest study describing lifestyle patterns in Latin America is the *Estudio Latinoamericano de Nutrición y Salud* (ELANS) (10), which analyzed food and nutrient intake as well as nutritional status and physical activity in more than 9,000 subjects (age range 15–65 years) living in urban locations across eight Latin American countries (i.e., Argentina, Brazil, Chile, Colombia, Costa Rica, Ecuador, Peru, and Venezuela). Overall, there is a low adherence to a high-quality diet, with limited consumption of diverse food groups, particularly in vulnerable subpopulations, but also with a reduced intake of micronutrients-rich foods, such as fruits and vegetables, legumes, and nuts (11). Only 7.2% of the overall sample reached World Health Organization (WHO)'s recommendation for fruit and vegetable intake (12). High percentages of the daily energy consumption came from high sugar and fat food sources, whereas only 18% was provided by food sources rich in fiber and micronutrients (13). These deficient food and nutrient intake-related habits present in Latin America are closely related to the pathogenesis and development of NCDs, which are explained by a dietary pattern deficient in nutrient-dense food groups as well as low levels of physical activity and a sedentary lifestyle (14, 15).

With regard to Chile, data from the 2016–2017 National Health Survey (16) showed an increasingly high prevalence of risk factors/conditions and NCDs: obesity: 34%; metabolic syndrome: 40%; blood hypertension: 28%; diabetes: 13%, and high CVD risk: 26%. Indeed, 27 and 25% of total mortality occurred in 2011 were attributed to CVD and cancer, respectively. Among environmental factors, we have reported low adherence to an abbreviated healthy dietary score, the Alternate Healthy Eating Index 2010 (AHEI-2010) (17), and to a locally adapted Mediterranean diet (MD) index (18) in our population. Furthermore, an alarming 98% prevalence of high-salt intake has been described (16). Overall, ongoing epidemiological trends and deficient food intake patterns summon implementation of effective nutritional and other lifestyle-based interventions to attenuate the escalating burden of NCD risk in Chile.

In this worldwide context of NCDs affecting developing regions, lifestyle medicine (LM) is a growing field (19) focused on overall promotion of healthy behaviors and its important role in prevention as well as treatment of and rehabilitation from disease. The most consensual definition of LM is “*evidence-based practice of assisting individuals and their families to adopt and sustain behaviors that can improve health and quality of life* (20). It works on six main axes: healthy eating, regular physical activity, avoiding risky substance abuse, stress management, optimizing good quality sleep, and promoting healthy social relationships. It is worthy noticing that a solid support in scientific evidence is a core foundation of LM, including its Hierarchies of Evidence Applied to Lifestyle Medicine (HEALM) (21) aimed to a better quality of backing from observational and interventional studies on the effects of lifestyle choices in public health and their long-term impact. Thus, effectiveness at preventing, managing, and sometimes reversing many NCD is a main issue in LM (22).

The American College of Lifestyle Medicine (ACLM) was founded in 2004 as an independent medical professional association representing the diverse interests of its members in providing of LM education, practice support, and advocacy. Then, the Lifestyle Medicine Global Alliance (LMGA) was initiated by the ACLM in 2015 in response to the increasing need for LM solutions in low- and middle-income countries and for communication and coordination between LM medical professional organizations around the world. Now, more than 20 sister organizations exist and are present in every continent, including the Chilean Society of Lifestyle Medicine (SOCHIMEV).

This worldwide multilateral endeavor is relevant because LM particularly addresses lifestyle habits and many of them have unique geographical, psychosocial, and cultural characteristics which differ from one country or world region to another. As a consequence, it must be emphasized that eating habits not only obey to the fulfillment of caloric and nutritional needs in general, but also to food production and availability as well as deep psychological and cultural roots given by geographic and economic variables according to various realities in human groups. Indeed, native people from Latin America have various ethnic origins. In particular, Chile exhibits a combination between native Americans, mostly descended from Mapuche aboriginals, and immigrants from Europe, which has created unique nutritional habits, culinary traditions, and social frameworks. Thus, it is important to consider local adaptability and feasibility of prescriptions and recommendations in LM initiatives, especially when promoting healthy eating at population and individual levels.

In this review, we cover the topic of healthy eating as one of the pillars of lifestyle medicine in a particular Latin American context. More specifically, we highlight the plant-based Mediterranean diet as a dietary pattern adaptable, applicable, and sustainable within the Chilean food, social, and cultural framework to address the current transition toward chronic diseases in our country.

## Plant-Based Food Intake Patterns as Healthy Diets

Food patterns can be defined as quantity, proportion, variety, and/or combination of different foods and beverages in diets as well as the daily/weekly frequency in which they are habitually consumed (23). Usually, a healthy diet is labeled as such when this set of foods offers adequate amounts of energy and nutrients necessary to maintain body functions in a context of physical and mental health. Therefore, this conceptualization of a healthy diet is far from referring to its use as a restrictive nutritional pattern for short-medium term in order to achieve a goal (fad diet). Instead, food intake patterns capture multiple dietary factors and provide a comprehensive assessment of diets, which should account for the complex interactions between nutrients and foods and their wide-ranging impact on human body composition and physiology.

Multiple studies have tried to answer the question on which is the best or healthiest diet (24–30). Therefore, it is important to recognize there is no single diet that is universally recognized for achieving the best clinical results in all people. Nor it is possible to attribute health benefits or harm to specific nutrients or foods directly and unequivocally in a cause-effect relationship. This uncertainty is secondary to limitations given by methodological factors in nutritional epidemiology and interventions. Second, people eat complex diets made up of many interdependent components rather than individual nutrients or foods (9). There are synergies among nutrients and foods present in different dietary patterns (31, 32). The degree of food processing can also influence their physical and chemical characteristics and subsequent health impact (33). Thus, dietary patterns may be more predictive of diet–disease associations than analyses focused on single foods or nutrients (34, 35).

Among various dietary patterns, high intake of mostly plant-based foods (mainly fruits, vegetables, nuts, oils, legumes, and whole grains) as well as low intake of animal products and ultra-processed foods has shown the strongest support from scientific evidence to demonstrate their human health benefits (36–39). Although plant-based diets are frequently associated with vegan/vegetarian diets, they entail a variety of eating patterns. Indeed, the expression “plant-based” is not intrinsically restrictive, but it means food intake mostly derived from plants, thus it may embrace consumption of small amounts of foods from animal origin such as meat, fish, milks, and eggs. Thus, a plant-based diet may exchange animal items for vegetable choices, but it does not require absolute and permanent restriction of animal foods. Then, it is necessary to emphasize that the concept of plant-based diet is an eating spectrum that does not represent a single specific all-or-nothing pattern, but rather a general outline with the common elements already described above, which at the same time allows tailoring it to the reality of each individual with a unique cultural context.

Therefore, it becomes evident why professionals dedicated to LM in Chile and around the world promote mostly whole-food plant-based diets -a term introduced a few years ago and recently popularized in Chile- as a fundamental pillar of a healthy diet (40). Leading proponents in the LM field have varying opinions about what comprises the optimal whole-food plant-based diet: from allowing small amounts or completely avoiding all animal-based foods as well as holding back nuts and soybeans, especially for coronary patients, where total fat and oils are generally restricted (41–43). However, there is a broad consensus on aiming to maximize the consumption of nutrient-dense plant foods in their whole form, especially vegetables, fruits, legumes, and seeds and nuts (the latter in smaller amounts), whereas limiting processed and animal products for maximal health benefits. Moreover, consumers are increasingly adhering to this trend. In fact, retail market for plant-based foods has grown faster than the overall retail food market and plant-based alternatives are expected to increase significantly due to health and sustainability issues (44, 45).

Thus, even though the main focus in nutrition has traditionally been on the distribution and proportion of dietary macronutrients, this new emphasis converges on the quality and proportion of micronutrients, phytochemicals, and antioxidants as well as the amount of fiber, highlighting a lower degree of food processing as a priority, and ensuring its variety and an optimal micronutrient profile without excess calories. The latter is key since in general all eating patterns associated with health (i.e., prevention of chronic diseases, quality of life, and longevity) are almost by definition based on moderate food intake and calorie restriction (46). In fact, high adherence to mostly plant-based food dietary indexes have been correlated with decreased chronic disease risk (47–50). The benefits of predominantly plant-based diets are also currently emphasized in overall health outcomes rather than preventing or treating individual pathological conditions, with a special accent on quality of life and long-term well-being, therefore not exclusively focused on a disease-oriented point of view.

There is also a greater understanding about the (patho)physiological effects of various dietary patterns. Recent research has highlighted the multiple interacting pathways linking diet, microbiome, and health (51–54) as well as the role of low grade, but sustained, inflammation in the development of many chronic diseases, mainly related to food-triggered metabolic effects (55–58). Indeed, some dietary patterns have been associated with inflammation. The Western dietary pattern has been linked with increased concentrations of inflammatory markers, whereas higher scores in the Healthy Eating Index and the Mediterranean diet as well as in plant-based diets are associated with lower concentrations of inflammatory indicators (59, 60). In recent years, it has even been proposed a dietary inflammatory index based on food groups to assess diet quality based on the pro- vs. anti-inflammatory potential (61).

In addition, big data omics approaches are being applied in nutritional research on plant-based diets leading to high throughput and more integrative perspectives (62–64). For instance, a specific metabolomic signature has been identified for the MD, not only reflecting adherence and metabolic response to this diet, but also predicting future CVD risk independent of traditional risk factors (65).

More recently, it has become increasingly important to understand and assess human feed habits with regard to their consonance with the environment on which we interdepend, incorporating the variable of environmental sustainability as well as the concepts of planetary health and One Health (66). A sustainable diet may be defined as “a diet comprised of foods brought to the market with production processes that have little environmental impact, is protective and respectful of biodiversity and of ecosystems, and is nutritionally adequate, safe, healthy, culturally acceptable, and economically affordable” (67). When scientific evidence on different dietary patterns is analyzed in terms of their contribution to environmental sustainability, plant-based diets are the ones that have the greatest support in minimizing carbon and water footprints (68), contributing favorably to people's and environmental health. Many studies have shown that reducing meat consumption can attenuate greenhouse gases while remaining nutritionally adequate (69, 70). Based on detailed calculations and projections, the EAT Lancet report proposed a diet that is allegedly sustainable, nutritious, and healthy. This model diet fits with a mostly plant-based food pattern, consisting of mainly vegetables, fruit, whole grain, legumes, nuts and unsaturated fats, only moderate to small amounts of fish and poultry, and no or very little red meat, processed meat, added sugars, refined cereals, and starchy vegetables. Adhering to this plant-based dietary recommendation, it should be possible to meet the United Nations Sustainable Development Goals (United Nations (UN), 2019) (71).

## Mediterranean Food Pattern: a Plant-Based Diet that Promotes Human and Planetary Health

Using the broad definition analyzed above, the traditional Mediterranean dietary pattern has been considered a mostly plant-based diet because it is aimed to a high intake of olive oil, fruit, nuts, vegetables, cereals, herbs, and spices; a moderate consumption of fish and poultry as well as wine with meals; and a low intake of dairy products, red meat, processed meats, and sweets.

The positive health outcomes associated with the MD were identified in the early 1960s, when researchers showed the protective effects against coronary heart disease of diets eaten in Southern Europe compared to Northern Europe and US (72). Since then, an increasing body of research has suggested the beneficial effects of this dietary pattern with evidence supporting its value on different clinical outcomes (73–76). Currently, the MD is considered one of the healthiest dietary patterns. Cross-sectional and prospective observational studies in Europe, US, and Australia have associated higher adherence to Mediterranean-style food patterns with lower prevalence/incidence of NCD conditions including diabetes, CVD, cancer, neurodegenerative diseases, and overall mortality (77–80).

The evidence regarding the association between the MD and cancer comes predominantly from observational studies. Most of these studies found a protective effect of high adherence to the MD against the development of different types of neoplasms, with a reduction in the risk of developing cancer in general ranging from 5 to 21% (36, 81). By types of cancer, risk reductions of 33% for gastric cancer (82), 36% for prostate cancer (83), 49% for hepatocellular cancer (84), 50% for colorectal carcinoma (85), 58% for head and neck cancer (86), 81% for lung cancer (87), and 6% in postmenopausal breast cancer (88) have been reported. The main limitation of these studies is that recording of dietary practices was done by means of quantified consumption frequency surveys or 24-h recall, whose high probability of bias makes it an unreliable tool. The only clinical trial with regard to cancer prevention is the PREDIMED study, being, so far, the one that provides the best quality of evidence demonstrating the benefit of this dietary pattern on cancer incidence, reporting a 67% reduction in breast cancer risk in women at high cardiovascular risk, when adhering to MD supplemented with extra virgin olive oil (89).

Among longitudinal studies, MD patterns have been linked with lower incidence of mental conditions, such as depression in Spain, Italy, US and Australia (90–97). Two additional trials (SMILES and MEAL) are evaluating the impact of implementing a MD pattern on depression prevention and/or treatment (98, 99). Interestingly, adherence to MD correlated cross-sectionally with self-esteem and self-concept in children living in Santiago (100). MD has also been proposed as one of the dietary patterns of choice for patients with chronic kidney disease (101).

It must also be taken into account the sex/gender-driven differences in metabolic response to nutrients (102, 103). Some dietary patterns have been specifically studied in women related health issues, for example higher consumption of vegetable protein has been associated with less risk of early menopause (104), and lower glycemic index diets have shown less rates of insomnia in postmenopausal women (105). As for the MD, to our extent of knowledge, there are certain studies that emphasize their results specifically in women, showing some associations with less risk of osteoporosis (106), and in an uncontrolled calorie-restricted MD, decreased serum levels of end glycation products were found in premenopausal women who had overweight or obesity (107). Also, the lower risk of incident stroke, mediated by a higher adherence to MD, appears to be driven specifically by an association found in women (108), and higher MD intake was associated with one-fourth relative risk reduction in CVD events in women from the Women's Health Study (109). In another study, MD was associated with less risk of rheumatoid arthritis only in men (110). Therefore, there is still more research needed in this field in order to confirm these sex-specific associations with MD intake as well to define their underlying mechanisms.

Far fewer interventional studies have also evaluated the effect of MD on long-term and hard clinical outcomes. First, the secondary prevention Lyon Heart Study exhibited a significantly lower recurrence of MI and reduced CVD mortality (111). Despite randomization issues recently addressed (112), PREDIMED (Prevención con Dieta Mediterránea) trial, a Spanish primary prevention study involving high cardiovascular risk participants, demonstrated that MD -without caloric restriction or physical activity recommendation but supplemented with olive oil or nuts- reduced CVD events by 30% compared to a low-fat diet (112, 113). Further analyses in PREDIMED have shown that MD attenuates diabetes mellitus incidence (114), diabetic retinopathy (115), age-related cognitive decline (116) and invasive breast cancer occurren (89). Whether reported irregularities in the randomization process (112) have influenced these latter findings reported by PREDIMED remain to be established. Interestingly, RCT study design in non-Mediterranean countries have been recently reported in US (117) and Australia (118).

Also, growing evidence from the so-called blue zones (119–123) -including two locations within the Mediterranean basin (e.g., Sardinia in Italy and Icaria in Greece)- exhibits long-lived and high life quality populations. Even though this non-scientific concept has yet to be better defined and further validated, it is interesting how these world regions seem to share some common elements -including consuming a predominantly plant-based diet- that have wide beneficial evidence on different outcomes in health.

As mentioned, MD relies on minimization of animal products and high consumption of a variety of fruits and vegetables, with legumes being a crucial part of this pattern, contributing also to soil health. Furthermore, MD emphasizes the choice of foods from local origin and seasonal production. For all this, there is consensus that to a greater or lesser degree, plant-based MD food choices align with planetary health (124, 125). It has even been estimated that switching to a MD can reduce greenhouse gas emissions, land use, energy consumption, and water utilization by up to 72, 58, 52, and 33%, respectively (126). Therefore, MD can contribute to increase the sustainability of food production and consumption systems in addition to its well-known benefits in disease prevention and public health.

## Mediterranean Diet as a Model of a Culturally Adaptable Food Consumption Pattern

As previously mentioned, diets are more than just the pattern of food consumption, representing also a way of life shaped by various economic, social, and cultural variables within the local context of each individual, including influences integrated through migration and globalization. Although they are closely linked to the biophysical resources (e.g., soils, microclimates, landscape) that characterizes agriculture, food patterns thus also take into account particular historical frameworks as well as sociocultural resources, including traditional knowledge, and practices (127).

Examples of regional/territorial diets are the Japanese Diet, the Traditional Nordic /New Nordic Diets and the MD, which incorporate in their definition not only the food pattern, but also cooking methods, celebrations, customs, lifestyle, and typical products of a region. As UNESCO highlighted when adding the MD to its list of Intangible Cultural Heritage of Humanity in 2010, it is “a set of skills, knowledge, practices and traditions from landscape to table, including crops, harvesting, fishing, conservation, processing, preparation and, in particular, food consumption” (128). While MD represents the dietary and overall lifestyle patterns of a small proportion of the global population, some of the basic principles that shape this diet, preference for local and seasonal foods, daily consumption of vegetables, fruits, whole grains, and healthy fats can be applied and adapted to other territories and cultures.

Moreover, the MD is not a strict dietary pattern, since foods show variations among countries within the Mediterranean basin. Indeed, it consists of a flexible dietary pattern that can be locally adapted based on food availability and cuisine traditions (129), which has led to being promoted in regions and dietary guidelines of countries far from its geographic origin.

## Mediterranean Diet: an Adaptable and Feasible Food Intake Pattern in Chile

Considering the particular Chilean context within Latin America, the MD model has shown to correspond to types of foods and culinary preparation that are part of the traditional eating culture in Chile. Remarkably, Chile is one of the five areas of the planet with a Mediterranean-type ecosystem, being its local agricultural, livestock, and aquaculture production very abundant in foodstuffs associated with the conventional MD (130, 131). Indeed, the majority of annual food exports from our country fits very well with a Mediterranean food basket (132). Using food availability data from the Food and Agriculture Organization (FAO) of the United Nations, the diet consumed by Chileans in the 1990s decade still showed similarities to the traditional MD of Spain and Italy in 1960 (133).

Moreover, Chilean culinary and gastronomic traditions apply food items and cooking methods that mimic those used in traditional cuisines from Southern Europe (134–137). Many dishes contain cooked vegetable and legume products and are prepared based on a sofrito. Likewise, tomato salad with onion and *pebres* are also mixtures rich in antioxidants and fiber, derived from the use of tomato, onion, garlic, parsley, chili pepper, cilantro, and vegetable oils. On the other hand, avocado is a typical food in our culinary culture, but not in the Mediterranean basin. However, it is characterized by a low contribution of saturated fats and high content in monounsaturated fatty acids as occurs in olives and olive oil. In addition, chestnuts, Chilean hazelnuts, and pine nuts are important in the Chilean aboriginal food culture. Indeed, pine nuts obtained from araucarias are considered the sacred fruit of the Mapuche indians.

When Mediterranean dietary culture first developed, typical families did not eat large amounts of meat and had no access to imported produce or foods out of season. Global food consumption levels and patterns have changed significantly, influenced among other factors, by population growth, urbanization, Westernization, and a rise in affluence and living standards (138). We are experiencing a nutritional transition in which problems of undernutrition coexist with overweight, obesity, micronutrient deficiencies, and food-related chronic diseases (5). Thus, it is important to understand drivers and barriers in consumer food choices and how these are shaped, since a greater adherence to a MD has been associated to its health outcomes (139–141) and also lower environmental pressure and impact (124–126, 142). Even though MD is a sustainable healthy diet, many people are unlikely to adhere to a food pattern if it is not culturally acceptable at population levels. In fact, there has already been a decline in adherence to the MD in the Mediterranean countries (143) and also in Chile (18).

At individual level, health professionals must fulfill the role of informing and advising based on their knowledge and applicability of MD-type nutritional recommendations to the context of each patient, without imposing beliefs, preferences, or ethical judgments. Therefore, it is essential to counsel taking into account personal, family, economical, and cultural circumstances as long as the basic criteria for plant-based MD healthy eating pattern described above are met. Also, LM moves broadly in a field going from prevention to therapy and reversal of chronic diseases. Therefore, lifestyle goals, including diet, must be individualized, and in the case of more complex chronic diseases, advised by specialists in each subject with solid professional training and balancing multiple variables.

Overall, we believe that the MD spectrum has great potential for its adaptation in our cultural context and may prove to be a feasible, affordable, and flexible model of healthy eating for the general Chilean population.

## Potential Health Impact of Mediterranean Diet Intake in Chile

As just discussed, promotion of MD adherence in Chile seems reasonable and doable offering a great potential for handling the increased prevalence of risk factors and NCDs in our country.

It is well-known that the most effective strategies for promoting long-lasting healthy habits that reduce the risk of NCDs, are the ones targeting the earliest ages along the human vital cycle (144–147). Therefore, it is important to create interventions to improve adherence to healthy diets considering the entire family, and specially targeting children and adolescents. Interestingly, there is an ongoing clinical trial (the Happy Heart) (148) evaluating the effectiveness of an educational program to improve healthy habits in children and their families, as compared to routine adult outpatient care. Also, it is worthy noticing that in Latin America, still much of what is eaten within the family context is driven by women decisions, therefore, strategies that account for their specific food choices and dietary habits remain important, in order to design effective and tailored interventions. Even more, targeting pregnant mothers is increasingly highlighted and has a lot of potential to be further explored, as it may influence the fetal programming of chronic diseases, and therefore, the offspring's health after birth (148–150). Indeed, a recently published study (151) showed that a higher adherence to a Mediterranean diet score and a lower dietary inflammatory index during pregnancy was associated to lower BMI z-score trajectories in the offspring from birth to adolescence.

However, the real health impact of implementing the MD remains to be evaluated appropriately in Latin America. Furthermore, limited and exploratory work has been executed to assess the impact of MD in associations studies or interventions in Chile. We have reported that just one-tenth of Chilean adults exhibited a high adherence score when applying a locally adapted and validated Chilean Mediterranean dietary index (18). Those highly adherent subjects showed cross-sectionally a lower prevalence of overweight, obesity, and metabolic syndrome (152), suggesting that improvement in adherence to MD may lead to significant reduction in these high-risk conditions. In small studies, MD implementation improved several biomarkers (e.g., blood fatty acid profile, haemostasis, oxidative stress, endothelial function, and advanced glycated products) (153–157) linked to NCD pathogenesis. In addition, an uncontrolled study aimed to mediterranize food availability at workplace showed that increasing adherence to MD was associated with reduction in abdominal obesity and blood pressure and increased HDL cholesterol levels, leading together to lower metabolic syndrome prevalence (158). Thus, evidence supporting health benefits of the MD in Chileans subjects shows potential, but studies available are scarce and have many limitations such as cross sectional or non-randomized controlled design, no control comparators for interventions, small sample size, lack of theory-based behavior change advice, short follow-up, biomarker-based end points only, and very limited clinical outcomes. Additional intervention studies using a locally adapted MD index, a feasible MD intervention, and a more comprehensive outcome evaluation are required –as an *in situ* proof of concept- to further emphasize and more extensively implement this dietary pattern for prevention and treatment of chronic diseases in our population.

More recently, a randomized clinical trial has been registered (ClinicalTrials.gov NCT03524742) to evaluate the effect of MD supplemented with avocado on LDL cholesterol levels in Chilean patients with high risk of recurrent ischemic stroke. In addition, we will perform the ChileMed study, a 1-year randomized controlled intervention that will assess the efficacy of a locally adapted MD -vs. a low-fat diet- for treatment of Chilean subjects with metabolic syndrome. MD adherence at baseline and follow-up will be evaluated with the Chilean MD index and 24-h dietary recalls as well as diet-derived biomarkers. Cardiometabolic risk parameters, including measures of inflammatory, oxidated stress, and metabolomic status, will be analyzed to elucidate possible mechanisms underlying a favorable outcome. Hopefully, these intervention studies will further support the beneficial impact of a plant-based MD approach in Latin America as expected from the ecosystem, food production and availability, culinary traditions, and cultural context of Chile.

## Conclusions

Abandonment of traditional habits and emergence of unhealthy lifestyles associated with socio-economic and cultural changes have become main threats to human health. Thus, promotion and preservation of healthy dietary patterns more aligned with our culinary and local traditions is crucial for a sustainable development to counteract food insecurity and malnutrition.

Overall, scientific evidence is consistently showing that a diet rich in various plant-based and whole foods is the pattern most associated with reduced morbidity, longevity, increased quality of life, and lower mortality.

In addition, plant-based dietary patterns are more sustainable food models that help balance and promote optimal planetary health. On the other hand, it is fundamental to use an individualized work model grounded to the sociocultural reality of each person and family. The most effective nutrition approaches are those that take into account cultural preferences and practices, rather than going against them.

We believe that the MD is a pattern well-aligned with mostly plant-based food intake as well as LM principles and practices and is strongly evidence-based. Therefore, it may be implemented and should be reinforced as an important tool at the level of public health policymaking beyond the Mediterranean basin. This could be particularly relevant and feasible in countries and word regions -such as Central Chile- where geography, food supply, gastronomy, and culture offer fertile soil to adopt and adapt this dietary pattern. Further research should help to substantiate this statement and to expand its implementation worldwide.

Even more, the definition of the MD not only includes food intake guidance, but it is also considered like a comprehensive lifestyle. Meal preparation, enjoying food with moderation and socially in a calm and relaxed environment, which together with the practice of regular physical activity, appropriate rest/sleep and positive psychosocial resources, community life, and a sense of belonging and sharing, are all part of the Mediterranean way of life (159).

Going back to LM work model, MD emphasizes within its definition not only healthy eating habits, but all of the other pillars of this emerging field that summons a variety of medical specialties and other health professions to promote human health and well-being.

## Author Contributions

CFi wrote the first draft of the manuscript. CFi, AR, XM, and GV wrote sections of the manuscript. All authors contributed to manuscript revision, read, and approved the submitted version.

## Conflict of Interest

The authors declare that the research was conducted in the absence of any commercial or financial relationships that could be construed as a potential conflict of interest.
